# Chondrotoxicity of Local Anesthetics: Liposomal Bupivacaine Is Less Chondrotoxic than Standard Bupivacaine

**DOI:** 10.1155/2020/5794187

**Published:** 2020-01-04

**Authors:** Travis Farmer, S. Craig Morris, Robert Quigley, Nirav H. Amin, Montri D. Wongworawat, Hasan M. Syed

**Affiliations:** ^1^Department of Orthopaedic Surgery, Loma Linda University, 11406 Loma Linda Drive, Suite 218, Loma Linda, CA 92354, USA; ^2^Department of Pediatric Orthopaedic Surgery, Children's Hospital at Erlanger, 910 Blackford Street, Chattanooga, TN 37403, USA; ^3^Department of Orthopaedic Surgery, VA Loma Linda Healthcare System, 11201 Benton Street, Loma Linda, CA 92357, USA

## Abstract

**Objective:**

The purpose of this study is to determine whether (1) liposomal bupivacaine is chondrotoxic; (2) the chondrotoxicity of liposomal bupivacaine differs from standard bupivacaine; and (3) chondrotoxic effects are time dependent.

**Materials and Methods:**

We obtained 72 10 mm articular cartilage plugs from 12 fresh bovine distal femoral knee joints and exposed them to either saline, 0.5% bupivacaine, or liposomal bupivacaine for either 30 or 90 minutes. Twenty-four hours after treatment, chondrocyte viability was measured with the use of a fluorescent live/dead assay. An ANOVA test of variance was performed followed by a Holm–Sidak test to make pairwise comparisons across conditions. Student's *t*-test was used to compare means.

**Results:**

Percent viability of cells exposed to liposomal bupivacaine for 30 minutes was less versus saline control (53.9% ± 21.5% vs. 73.7 ± 18.4%, *p*=0.035), and this remained significant at 90 minutes (49.1% ± 20.3% vs. 67.2% ± 25.6%, *p* < 0.001). Liposomal bupivacaine had less chondrotoxic effects when compared with bupivacaine after 90 minutes, with greater viability (49.1% ± 20.3% vs. 21.4% ± 14.0%, *p*=0.003). Chondrotoxicity was found to be time dependent within the bupivacaine group (percent viability at 30 min: 45.5 ± 18.2%, 90 min: 21.4 ± 14.0%, *p*=0.001); however, liposomal bupivacaine did not demonstrate a significant time-dependent chondrotoxic relationship (*p*=0.583).

**Conclusions:**

Bupivacaine and liposomal bupivacaine are both toxic to chondrocytes. Liposomal bupivacaine is less chondrotoxic than standard bupivacaine and does not demonstrate a time-dependent toxicity.

## 1. Introduction

Intra-articular injections of local anesthetics are commonly used for managing pain associated with degenerative joint disease, as well as pain associated with arthroscopic procedures. They have been used for many years and have been shown to be efficacious in the ambulatory setting for diagnostic and therapeutic purposes. Administration of intra-articular local anesthetics during the perioperative period has also been shown to improve postoperative pain scores and reduce opioid consumption and opioid-related adverse events such as respiratory depression, sedation, and constipation [1]. Concern has risen regarding complications associated with intra-articular injections particularly in arthroscopic procedures involving healthy cartilage. Investigators have found that bupivacaine and other local anesthetics are toxic to chondrocytes, and in vitro and in vivo studies have shown that even brief exposures result in decreased chondrocyte metabolism, increased chondrocyte apoptosis and necrosis, and gross morphologic cartilage degradation [2–8]. Pain pump models have shown gross cartilage necrosis after sustained exposure to local anesthetic infusion [9]. However, the extent of chondrotoxicity between local anesthetics varies with some demonstrating more chondrotoxicity than others [5, 9–11].

Local anesthetics injected into a joint provide known analgesic benefits, and for this reason, there is a continuing search for less chondrotoxic formulations. A novel formulation of liposomal bupivacaine was approved by the FDA in October 2011. It consists of multivesicular liposomes that release bupivacaine slowly over a longer period of time than standard bupivacaine. The half-life of liposomal bupivacaine is approximately 34 hours [1] compared with 2.7 hours for standard bupivacaine. This allows for sustained pain relief after operative or ambulatory procedures. The purpose of this study is to determine whether (1) liposomal bupivacaine is chondrotoxic; (2) the chondrotoxicity of liposomal bupivacaine differs from standard bupivacaine; and (3) chondrotoxic effects are time dependent. Our null hypothesis was that liposomal bupivacaine was neither chondrotoxic nor had time-dependent toxicity.

## 2. Materials and Methods

**Figure 1:**
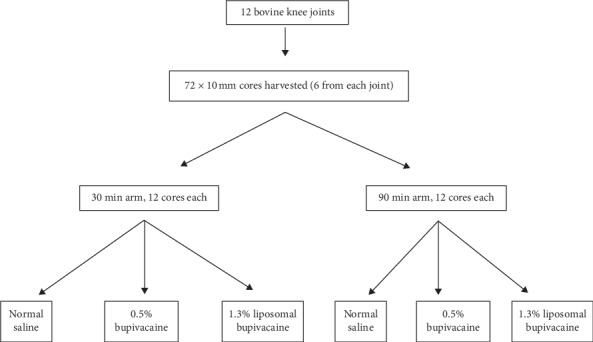
Flow diagram depicting articular plug division into study groups.

We collected articular cartilage from 12 freshly slaughtered bovine knee joints. Typical age at euthanasia at a local abattoir is approximately 18 months for beef cattle. Bovine cartilage is an accepted in vitro cartilage model and has been used in previous local anesthetic studies [3, 4, 6]. We harvested 6 articular plugs (3 from each condyle) from the distal femoral articular surface of each knee for a total of 72 cores, using an OATS harvester system (Arthrex, Naples, FL) and obtained 10 mm cores. The plugs were maintained in tissue culture media (Dulbecco's Modified Eagle Medium/F12, 10% fetal bovine serum, 1% penicillin/streptomycin, and 1% fungizone) and, within 4 hours of procurement, exposed them to the experimental treatments. The plugs were then randomly separated into 6 groups of 12 cores each. The groups were then submerged in either normal saline, 1.3% undiluted liposomal bupivacaine (Exparel®, Pacira Pharmaceuticals, Parsippany, NJ, USA), or 0.5% bupivacaine. The concentration of liposomal bupivacaine was chosen as this is the commercially available concentration. Standard formulation bupivacaine 0.5% was chosen as this is a common clinically used concentration. Also, for comparison, other studies have used this concentration for studying chondrotoxicity of bupivacaine [2–4, 7, 9–12]. Three groups of plugs (*n* = 12 each) were submerged for 30 minutes, and three groups of plugs were submerged for 90 minutes (Figure 1).

**Figure 2:**
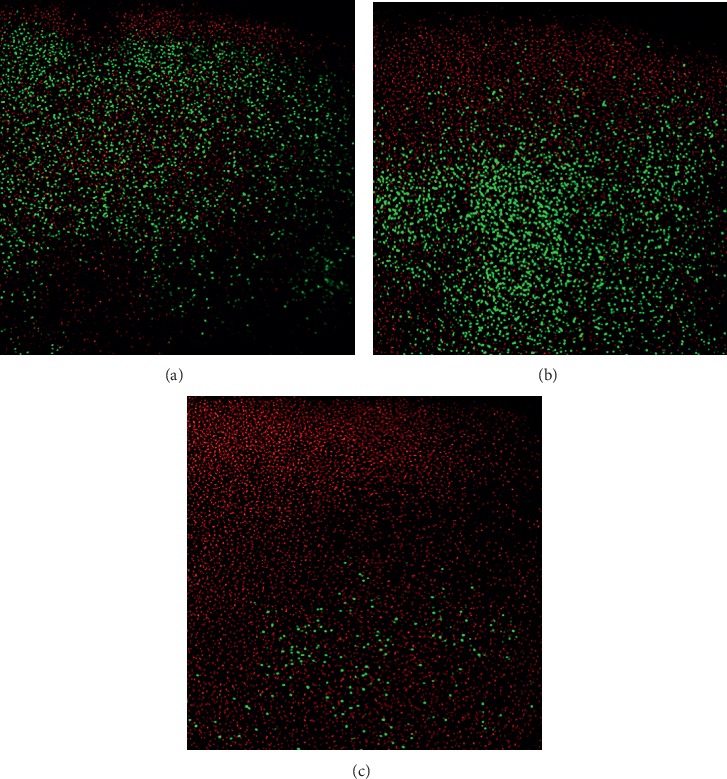
Sample articular cartilage plugs confocal microscopy stained with live/dead assay is shown after 24 hours of 90 minute exposure using (a) saline, (b) liposomal bupivacaine, and (c) standard bupivacaine. Live cells appear green, while dead cells appear red.

The plugs were then washed with normal saline before transferring them to new tissue culture media. After 24 hours, we used a vibratory microtome (Vibratome, St Louis, MO, USA) to obtain one 100 *μ*m thick slice from the middle third of each articular plug and oriented perpendicular to the articular surface. Each slice was then stained with 5 *μ*M of 5-chloromethylfluorescein diacetate (CMFDA; Molecular Probes, Eugene, OR) and 1.5 *μ*M of propidium iodide for 30 minutes at room temperature in the dark. 5-chloromethylfluorescein diacetate is a commercially available green fluorescent dye that freely passes through cell membranes where it is enzymatically transformed into an impermeable molecule. Propidium iodide, however, is membrane impermeable, and when bound to nucleic acids, it fluoresces red and is commonly used to identify nonviable cells. Stained sections were then washed with phosphate-buffered saline (PBS). Images were taken using a Zeis Axio Observer Z1-inverted LSM 710 NLO laser scanning confocal microscope with a water immersion X63 C Plan-Apochromat (NA 1.2) objective. Images were of 1.661 × 1.661 *μ*m and were of 1024 × 1024 pixels, with a 16 bit sampling depth. Different lasers were used to excite each dye. 5-CMFDA was activated with a 488 nm laser, and propidium iodide was activated with a 561 nm laser. The image was taken by visual inspection of the peak fluorescence signal. The images were saved and analyzed using a public domain Java image processing program (ImageJ; National Institutes of Health, Bethesda, MD, USA). To count the number of chondrocytes, others had quantified the size of each chondrocyte and the chondrocyte nucleus [13]. We used standard-sized beads on confocal microscopy to determine the particle size of either a live or dead chondrocyte or chondrocyte nucleus. We then used histogram analysis and standard deviation of the particle size to determine the number of chondrocytes on each confocal image, either green for live cells or red for dead cells (Figure 2).

An ANOVA test of variance was performed followed by a Holm–Sidak test to make pairwise comparisons across conditions to compare the chondrocyte viability for the means of the different treatment arms. Within groups, Student's *t*-test was used to compare mean viability. Results are presented as the percentage of live chondrocytes (percent viability).

## 3. Results

When compared with saline control, percent viability in plugs exposed to liposomal bupivacaine at 30 minutes was less (73.7 ± 18.4% vs. 53.9% ± 21.5%, *p*=0.035). Liposomal bupivacaine continued to show a statistical reduction in viable cartilage cells compared with normal saline at 90 min (49.1% ± 20.3% vs. 67.2% ± 25.6%, *p* < 0.001) (Table 1). Standard formulation of bupivacaine demonstrated significant chondrotoxicity at all time points compared with saline (Table 1).

Liposomal bupivacaine was significantly less chondrotoxic than bupivacaine at 90 minutes (49.1% ± 20.3% vs. 21.4% ± 14.0%, *p*=0.003). However, liposomal bupivacaine did not demonstrate a significant difference in chondrocyte viability compared with standard bupivacaine at the 30-minute time point (53.9% ± 21.5% vs. 45.5% ± 18.1%, *p*=0.305). Standard formulation bupivacaine demonstrated significant chondrotoxicity at all time points compared with saline (Table 1).

**Figure 3:**
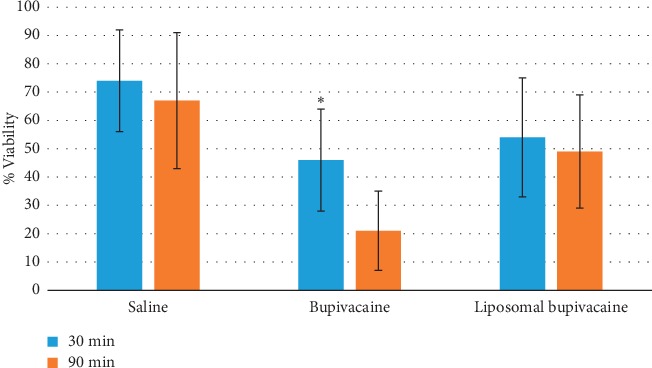
Percent viability of chondrocytes for each experimental group at 30 min and 90 min.

Chondrotoxicity was found to be time dependent for bupivacaine, but liposomal bupivacaine did not reach statistical significance (Figure 3). Liposomal bupivacaine percent viability was not different between 30 minutes and 90 minutes (53.9% ± 21.5% vs. 49.1% ± 20.3%, *p*=0.583). Bupivacaine chondroxicity was greater after 90 minutes than after 30 minutes (21.4% ± 14.0% vs. 45.5% ± 18.1%, *p*=0.001).

## 4. Discussion

The primary outcomes of our study were to determine whether liposomal bupivacaine was chondrotoxic, whether it was more chondrotoxic than standard formulation bupivacaine, and if chondrotoxicity was time dependent. We found that liposomal bupivacaine was in fact chondrotoxic in a time-dependent manner, rejecting our null hypothesis. Moreover, liposomal bupivacaine was less chondrotoxic than bupivacaine at 90 minutes.

Reports of postoperative glenohumeral chondrolysis have raised concern regarding the potential chondrotoxicity of local anesthetics [14, 15]. Previous in vitro studies have shown that bupivacaine and lidocaine may be chondrotoxic. A novel formulation of liposomal bupivacaine has recently been introduced, but there is a paucity of data published regarding the safety of intra-articular liposomal bupivacaine. Shaw et al. harvested bovine knee cartilage and isolated the chondrocytes in the medium prior to exposure to liposomal bupivacaine, bupivacaine, and ropivacaine [16]. Their results showed that after a 1-hour exposure to liposomal bupivacaine, chondrocyte viability was similar to the control. Liposomal bupivacaine showed higher chondrocyte viability compared with ropivacaine and bupivacaine. In their study, chondrocyte viability was much greater which is most likely due to higher baseline cell viability. They used first-passage chondrocytes only, suggesting that the study started with a healthier and more homogeneous population. In comparison with our study, we exposed articular plugs rather than chondrocyte medium to local anesthetic which may be more representative of in vivo exposure. In contrast, our study showed reduced viability of chondrocytes in the liposomal bupivacaine group compared with control. Moreover, Shaw et al. reported results at only one time point; therefore, time-dependent toxicity could not be assessed.

Sridaran et al. studied human chondrocyte exposure to liposomal bupivacaine [17]. They harvested macroscopically normal articular cartilage from adult patients undergoing ankle fusions and exposed them to lyophilized bupivacaine resuspended in ethanol, bupivacaine at varying formulations, liposomal carrier DepoFoam (Pacira), and liposomal bupivacaine. Similarly, they found decreased viability with bupivacaine compared with liposomal formulation. They noted a time-dependent toxicity of liposomal bupivacaine over the course of 6 hours.

Our study concurred with previously published literature demonstrating that liposomal bupivacaine is also chondrotoxic, yet this study aids in quantifying how toxic liposomal bupivacaine is to chondrocytes. Previous studies have shown that all local anesthetics are chondrotoxic to some degree [2, 5, 7, 14]. However, there is a significant variation in the degree of chondrotoxicity between local anesthetics, and thus studying a novel formulation is valuable in determining the degree of chondrotoxicity and further understanding the time-dependent nature of that toxicity.

Liposomal bupivacaine, however, was shown to be less toxic than the standard bupivacaine preparation after 90 minutes. Liposomal bupivacaine may have less chondrotoxicity due to a lower peak concentration (1 : 6), a longer time to peak concentration (1 : 12), and a longer half-life than standard bupivacaine (36 : 3.5) [1]. The potential benefits of having a lower concentration of bupivacaine in the joint for a longer period of time, for extended pain relief, must be weighed with the risks of chondrocyte toxicity. These data suggest liposomal bupivacaine may be a better choice for intra-articular injection than bupivacaine, providing a longer period of pain relief with less chondrotoxicity over time.

Our study showed that the effects of standard formulation bupivacaine on articular cartilage continue with the length of time of exposure. Bupivacaine was more chondrotoxic at 90 minutes than at 30 minutes. This concurs with previously published studies which have demonstrated the time-dependent chondrotoxicity of local anesthetics, including lidocaine, bupivacaine, and ropivacaine, as well as steroids [6, 10, 12]. Interestingly, liposomal bupivacaine did not significantly differ in chondrotoxicity at 90 and 30 minutes. The liposomal formulation may provide a time-dependent protection through its pharmacokinetic differences.

Ropivacaine is another long-acting local anesthetic with similar duration of activity as bupivacaine [18]. The literature on ropivacaine chondrotoxicty has been variable, with some studies showing no chondrotoxicty after 30 minutes of incubation in human chondrocytes [7] or 12 hours [5]. Other studies showed chondrotoxicty at higher concentrations of 0.75% [19]. Shaw compared rovicapaine with liposomal bupivacaine, demonstrating higher viability of chondrocytes in the liposomal group [16].

This study has several limitations. Our study was performed in vitro, and the observations cannot necessarily be applied directly in vivo; therefore, all results need to be confirmed clinically. Our study used bovine cartilage and not human cartilage, and therefore the conclusions drawn from this study may or may not be applicable to humans. However, many of the previous studies on this subject have been done on bovine cartilage [3, 4, 6, 14].

We used one concentration of each experimental agent and only two time points, and as such our findings may be difficult to apply to in vivo conditions given the potential dilutional effects in the joint. We did not directly assess the free bupivacaine concentration in the experimental solution containing liposomal bupivacaine. Because the dissolution of liposomes, both in vivo and in vitro, depends on multiple factors including pH, volume, and vascularity, it is impossible to control the diffusion of the bupivacaine. As such, we attempted to model the in vivo environment in vitro and study the spontaneous diffusion of bupivacaine. Longer duration incubation would have been ideal; however, the in vitro nature of this study presents inherent limitations. The cartilage has been explanted from the live animal, and as such there is increased fragility of the cells and increased susceptibility to any experimental agent. Our suspicion was that we would obtain confounding data at longer time intervals due to the natural degradation of chondrocytes in an in vitro model. The 90-minute time point was also chosen based on previous published studies with multiple time point incubation periods [6, 10]. A potential future study may be to examine longer time points and the effects on the cartilage of these same formulations.

The lipid-based depot (DepoFoam) used in the liposomal group consists of multivesicular liposomes with nonconcentric multiple lipid layers [20]. This structure has been suggested to result in an increased level of stability and longer duration of drug release. While the lipid-based depot in isolation without medication could have served as an appropriate control but was unavailable for testing. The basic structure of the depot, however, prevents the depot from interacting with the cartilage membrane or entering into cells. As such, saline was felt to be an appropriate alternative control medium in an attempt to match the in vivo environment.

Compared with previously published studies, however, we believe our study has several strengths. First, our study used osteochondral cores of articular cartilage. There have been previous studies looking at the value of having intact cartilage matrix versus isolated chondrocytes, as the articular surface acts as a natural barrier to medications and therefore is a more reliable model. Second, we used a protocol similar to that used in previous studies, which adds to the validity of our study methods and also facilitates comparison of our results with prior studies. While additional anesthetics could have been useful to compare with liposomal bupivacaine, by comparing only standard formulation bupivacaine, we have been able to identify the differences based solely on the liposomal formulation.

## 5. Conclusions

Similar to other anesthetics, liposomal bupivacaine is toxic to chondrocytes; however, it is less chondrotoxic than standard bupivacaine and may be safer for intra-articular analgesia given less time-dependent toxicity. The clinical relevance of exposure duration and its effects is not known, and further study on specific dose- and time-dependent effects is warranted.

## Figures and Tables

**Table 1:** Chondrotoxicity results across experimental groups.

Percent viability	Normal saline	0.5% bupivacaine	1.3% liposomal bupivacaine	1.3% liposomal bupivacaine vs. 0.5% bupivacaine
30 minutes	74 ± 18%	46 ± 18% *p*=0.004^a^	54 ± 21% *p*=0.035^a^	*p*=0.305
90 minutes	67 ± 24%	21 ± 14% *p* < 0.001^a^	49 ± 20% *p*=0.034^a^	*p*=0.003

^a^ *p* value reported against saline control.

## Data Availability

The percent viability data used to support the findings of this study are available from the corresponding author upon request.
